# Structural basis of *Staphylococcus epidermidis* biofilm formation: mechanisms and molecular interactions

**DOI:** 10.3389/fcimb.2015.00014

**Published:** 2015-02-17

**Authors:** Henning Büttner, Dietrich Mack, Holger Rohde

**Affiliations:** ^1^Institut für Medizinische Mikrobiologie, Virologie und Hygiene, Universitätsklinikum Hamburg-EppendorfHamburg, Germany; ^2^Mikrobiologie/Infektiologie, Bioscientia Labor Ingelheim, Institut für Medizinische Diagnostik GmbHIngelheim, Germany

**Keywords:** *Staphylococcus epidermidis* biofilm formation, regulation, PIA, Aap, Embp, AtlE, primary attachment, biofilm accumulation

## Abstract

*Staphylococcus epidermidis* is a usually harmless commensal bacterium highly abundant on the human skin. Under defined predisposing conditions, most importantly implantation of a medical device, *S. epidermidis*, however, can switch from a colonizing to an invasive life style. The emergence of *S. epidermidis* as an opportunistic pathogen is closely linked to the biofilm forming capability of the species. During the past decades, tremendous advance regarding our understanding of molecular mechanisms contributing to surface colonization has been made, and detailed information is available for several factors active during the primary attachment, accumulative or dispersal phase of biofilm formation. A picture evolved in which distinct factors, though appearing to be redundantly organized, take over specific and exclusive functions during biofilm development. In this review, these mechanisms are described in molecular detail, with a highlight on recent insights into multi-functional *S. epidermidis* cell surface proteins contributing to surface adherence and intercellular adhesion. The integration of distinct biofilm-promoting factors into regulatory networks is summarized, with an emphasis on mechanism that could allow *S. epidermidis* to flexibly adapt to changing environmental conditions present during colonizing or invasive life-styles.

## Medical relevance of *Staphylococcus epidermidis*

*Staphylococcus epidermidis*, member of the group of coagulase-negative staphylococci, belongs to the commensal skin flora of every human individual (Kloos, 1980, 1997; Kloos and Schleifer, 1986). In its natural niche, the species is of significant importance for maintenance of local homoeostasis—a role that is so far understood only superficially (Grice and Segre, 2011). Only recently light was shed onto the potential importance of *S. epidermidis* to interfere with colonization with *Staphylococcus aureus* via expression of a serine-type protease termed Esp, thereby interfering with colonization mechanisms of *S. aureus*, e.g., biofilm formation (Iwase et al., 2010; Sugimoto et al., 2013). Given the tremendous abundance of *S. epidermidis* on the skin, it is not surprising that *S. epidermidis* is the most common cause of contamination in clinical specimens, and it is a challenge for medical microbiologist to reliably identify true invasive isolates (Mack et al., 2006). Improved abilities to discriminate between clinically relevant and contaminating *S. epidermidis* isolates is of utmost importance, as the species is today one of the most important bacteria related to hospital acquired infections. If the invasive behavior of *S. epidermidis* follows a clear pathogenic strategy that extends its colonizing abilities (Mack et al., 2009), or merely can be regarded as an accident during which mechanisms usually contributing to commensalism turn into virulence features (Otto, 2009), is still a matter of debate. Nevertheless, without doubt *S. epidermidis* is a true opportunistic pathogen that only causes disease in patients with predisposing factors. These include individual patient characteristics (i.e., premature birth, congenital immune defects) or concomitant medical conditions—like HIV infection, immunosuppression after bone marrow or solid organ transplantation and chemotherapy related neutropenia (Goldmann and Pier, 1993; Rupp and Archer, 1994). Most significantly, *S. epidermidis* is the leading organism isolated from foreign material related infections (FMRI) (Darouiche, 2004; Geipel and Herrmann, 2005) such as infected prosthetic joints, central venous catheters (CVC), cerebrospinal fluid shunts, intracardiac devices, artificial heart valves, and vascular grafts (Mack et al., 2006; Rogers et al., 2009). Use of implanted medical devices increases in number, and certainly, this will further propel the importance of *S. epidermidis* as an important human pathogen.

*S. epidermidis* is responsible for the vast majority of nosocomial catheter related blood stream infections (CRBSI) in the United States (Wisplinghoff et al., 2003; Hidron et al., 2008) and also in German intensive care units (ICUs) (Geffers and Gastmeier, 2011). Evaluation of a multicenter international data collection calculated a risk of 6.8 CRBSI per 1000 central line-days in ICUs (Rosenthal et al., 2014). Results from the Surveillance and Control of Pathogens of Epidemiological Importance (SCOPE) indicate that coagulase-negative staphylococci are the most frequent cause of nosocomial blood stream infections (Wisplinghoff et al., 2003). Species discrimination identified eighty percent of CoNS from these infections as *S. epidermidis* (Jukes et al., 2010).

CoNS rank as third most common infective agent in native (NVIE) and first in prosthetic valve infective endocarditis (PVIE) clearly demonstrating the importance of CoNS in these clinical entities (Murdoch et al., 2009). Among CoNS isolates, about eighty percent were identified as *S. epidermidis* (Chu et al., 2004, 2009). Evaluation of the results of the International Collaboration on Endocarditis database showed a significantly higher rate of complicated clinical courses of PVIE due to CoNS with respect to heart failure compared to *S. aureus* or viridans streptococci (Lalani et al., 2006).

*S. epidermidis* is a significant cause of infections of prosthetic joint implants. The lack of non-invasive curative treatment options for joint implant infections often necessitates surgical intervention including replacement surgery. In the UK, CoNS and *S. epidermidis* were isolated in 36% of total hip and 49% of total knee arthroplasty infections (Phillips et al., 2006; Nickinson et al., 2010). In another study of infected total hip and knee arthroplasties about 77% of the isolated CoNS were confirmed *S. epidermidis* (Rohde et al., 2007).

First evidence suggesting a pathogenetic link between foreign-material implantation and *S. epidermidis* infection came from early electron microscopic analysis of explanted central venous catheters. Here, bacteria were found to colonize artificial material in large agglomerations, embedded into an amorphous material (Peters et al., 1982). While this specific mode of growth was first referred to as “slime,” today it is termed biofilm formation (Götz, 2002). In fact, there is significant evidence connecting the biofilm mode of growth to the general persistent course of *S. epidermidis* foreign-material infections (Scherr et al., 2014a), and to the regular failure to eradicate infections by antimicrobial therapies (Lewis, 2005; Mack et al., 2009; Otto, 2009; Rohde et al., 2010). The latter aspect has been subject to extensive review recently (Lewis, 2010). Although *S. epidermidis* infections are regarded as prototypic biofilm infections (Costerton et al., 1999; Otto, 2009), it must be noted that it is by far not clear that biofilms observed under *in vitro* conditions indeed correlate with the biofilm growth evident *in vivo*. While this is most probably the case in central venous catheter (Peters et al., 1981) or cerebrospinal fluid shunt (Kockro et al., 2000) infections, there is some doubt that this model can be easily transferred to infections occurring at the interface of an implant and the surrounding tissues, e.g., prosthetic joint infections (Broekhuizen et al., 2008; Zaat et al., 2010). Clearly, much needs to be learned with regard to the exact spatial organization of *S. epidermidis* in implant infections, e.g., by making use of *ex vivo* or *in vivo* imaging approaches.

## Structural factors contributing to *S. epidermidis* biofilm formation

Traditionally, the process of biofilm formation is divided into at least three steps. During the phase of primary attachment, bacteria adhere to the surface to be colonized, while during the accumulative phase, bacteria initiate the establishment of a three dimensional, multi-cellular and multi-layered architecture in which, intriguingly, most bacteria do not have direct contact to the surface (Mack et al., 2009; Otto, 2009; Rohde et al., 2010). *S. epidermidis*, then, is able to disassemble the biofilm structure again, and liberated cells are believed to allow *S. epidermidis* to colonize additional body sites. The process of biofilm formation needs a wide range of functional activities, ranging from molecules mediating binding to native or conditioned (i.e., host extracellular matrix covered) surfaces, over glue-like factors fostering cell-cell aggregation, to activities that break down matrix components (Otto, 2009). The tremendous diversity of specific functional requirements during biofilm formation is on the bacterial side significantly mirrored by the expression of a plethora of different, highly specialized factors characterized by very distinct profiles of biological functions. Thus, related to their specialized functions during *S. epidermidis* biofilm morphogenesis, specific factors are assigned to groups representing mechanisms being active either during primary attachment or during biofilm accumulation, respectively.

## Factors involved in primary attachment

Tight binding of bacteria to foreign-materials is a pivotal step toward establishment of a device-associated infection. Not unexpectedly, factors specifically involved in mediating bacterial–surface interactions were identified and further characterized. Some genetic evidence suggests that bacterial binding to unmodified polystyrene is fostered by the *S. epidermidis* autolysin AtlE (Heilmann et al., 1997). AtlE is a 115 kDa protein, which belongs to a group of bacterial peptidoglycan (PGN)-hydrolases playing a pivotal role in the degradation of the bacterial cell wall (Biswas et al., 2006). The protein consists of an N-terminal signal peptide, a propeptide, a catalytic domain with *N*-acetylmuramyl-L-alanine amidase activity, three repeats (R1–3), and a C-terminal catalytic domain with *N*-acetylglucosaminidase activity (Schlag et al., 2010; Zoll et al., 2010). In addition to its general role in cell wall turnover, AtlE also is of importance for binding to unmodified polystyrene, as demonstrated by the defect of an *atlE*::Tn*917* transposon mutant of *S. epidermidis* O-47 that lost its ability to adhere to plastic surfaces (Heilmann et al., 1997). Intriguingly, the importance of distinct domains for recruitment of AtlE to the bacterial cell wall (i.e., internal repeats) and enzymatic activities have been identified (Zoll et al., 2010). High resolution structural information is available, however so far it remains unclear, which exact AtlE domains are relevant for the primary attachment process and stable bacterial surface binding. In fact, at present it appears possible that expression and functional activation of AtlE induces significant changes in cell surface hydrophobicity, and thus, the AtlE effect on primary attachment might be secondary (Otto, 2014). In addition, a significant role of AtlE in eDNA mediated *S. epidermidis* biofilm formation is apparent (Qin et al., 2007; Christner et al., 2012).

While interactions between *S. epidermidis* and unmodified artificial surfaces most likely does not involve specific receptor-ligand interactions, it is well-known that *S. epidermidis*, similar to *S. aureus*, expresses cell surface proteins that mediate specific interactions with host extracellular matrix (ECM) components (Patti et al., 1994). Proteins with ECM-binding activity are believed to be of significant importance for the initiation of a device infection, since foreign materials become, as soon as they are inserted into the body, covered by ECM material (e.g., FN, fibronectin; Fg, fibrinogen; Vn, vitronectin; Cn, collagen) (Arrecubieta et al., 2006; Mack et al., 2009). In fact, *S. epidermidis* can use AtlE to adhere to surface organized Vn, while the lipase GehD is involved into interactions with collagen (Bowden et al., 2002). In addition to these proteins, for which their enzymatic activities might be of primary importance for *S. epidermidis* physiology, *S. epidermidis* also expresses proteins with a primary and dedicated function in bacterial-ECM interactions. These proteins belong to the group of serine-aspartate repeat (Sdr) proteins (McCrea et al., 2000), a widely investigated protein family of microbial surface components recognizing adhesive matrix molecules (MSCRAMM) (Josefsson et al., 1998; Foster et al., 2014). In *S. epidermidis*, three Sdr proteins referred to as SdrF, SdrG, and SdrH have been identified (Josefsson et al., 1998). SdrG, a LPXTG-motif containing protein covalently attached to the bacterial cell surface, is crucial for *S. epidermidis* adherence to fibrinogen-coated surfaces. It is therefore also being referred to as fibrinogen binding protein of *S. epidermidis* (Fbe) (Nilsson et al., 1998; Pei et al., 1999; Hartford et al., 2001). The gene encoding Fbe/SdrG is common in clinical *S. epidermidis* isolates (Nilsson et al., 1998; Rohde et al., 2004, 2007). Fbe/SdrG protein contains five distinct regions: an N-terminal export motif sequence, an A region that contains the Fg binding activity, a B region of so far unknown function, and the R region containing serine-aspartate repeat sequences. Fbe/SdrG specifically binds to a peptide sequence of 14 amino acids found in the N-terminus of the β-chain of Fg (Ponnuraj et al., 2003), and structural analysis of the interaction revealed a unique “dock, lock, and latch” mechanism ensuring a particularly strong interaction (Bowden et al., 2008; Herman et al., 2014). SdrF, sharing overall organizational similarity with SdrG, has been shown to mediate *S. epidermidis* binding to collagen I (Arrecubieta et al., 2007). In contrast to Fg-binding properties of Fbe/SdrG, the collagen binding epitopes of SdrF are located within the B repeat region (Arrecubieta et al., 2007). So far no specific functionality has been attributed to the N-terminal A domain of SdrF. A single B domain repeat of SdrF was sufficient to interact with collagen I, and apparently, this binding occurs via interactions with the α1 and α2 chains of type I collagen (Arrecubieta et al., 2007). Using a *Lactococcus lactis* heterologous expression system and a murine infection model evidence was generated that SdrF may contribute to cardiac assist device driveline infections (Arrecubieta et al., 2009). SdrF also mediates binding to unmodified Dacron surfaces covering drivelines. In contrast, *L. lactis* expressing GehD bound only weakly to driveline surfaces (Arrecubieta et al., 2009). Anti-SdrF inhibited *S. epidermidis* 9491 binding in the *in vivo* model only by roughly 50%, indicating that additional *S. epidermidis* collagen binding factors may be involved (Arrecubieta et al., 2009).

Extensive work has addressed the role of extracellular DNA (eDNA) in *S. epidermidis* and *S. aureus* biofilm formation. Data confirm that eDNA is a structural component of the biofilm matrix in both species, although evidence anticipates that eDNA has, at least partially, different functions in both species. Several independent studies have demonstrated that eDNA is released through increased cell lysis (Allesen-Holm et al., 2006; Rice et al., 2007; Christner et al., 2012). In *S. epidermidis* autolysis is determined to a large extent by the activity of the major autolysin AtlE (Biswas et al., 2006). A role for eDNA in *S. epidermidis* 1457 during primary attachment was deduced from observations showing that addition of DNase I abrogated bacterial attachment to glass surfaces. These findings were confirmed in additional, genetically independent *S. epidermidis* backgrounds (i.e., RP62A) (Qin et al., 2007). In extent to its effect on primary attachment, eDNA functions as an intercellular adhesin contributing to the stabilization of biofilms (Whitchurch et al., 2002). Based on the finding that DNase I has biofilm disintegrating activity when added within the first 6 h of biofilm accumulation currently it is believed that eDNA mediated intercellular adhesion is critical especially during the early accumulative phase (Qin et al., 2007). A role of eDNA in earlier stages of staphylococcal biofilm formation has recently been underpinned by observations showing that during *S. aureus* surface colonization under flow conditions eDNA, while not having impact on primary attachment, is critical during the transition from attachment to accumulation (Moormeier et al., 2014). It should again be stressed that functional differences of eDNA during *S. epidermidis* and *S. aureus* biofilm formation are apparent (especially with respect to the function during accumulation) (Izano et al., 2008; Christner et al., 2012), and observations in one species cannot easily be extrapolated to the other. This is especially true for the role of eDNA as a target during biofilm detachment events. By *saeRS* regulated expression of nuclease Nuc *S. aureus* can remodel the biofilm ultrastructure and control the release of bacteria from established biofilms (Mann et al., 2009; Olson et al., 2013). The lack of nuclease activity questions if this biofilm-escape mechanism is, in addition to *agr*-mediated biofilm dispersal (Vuong et al., 2003; Wang et al., 2011a), of relevance in *S. epidermidis*.

## Mechanisms of *S. epidermidis* biofilm accumulation

The hallmark of the accumulative phase is expression of intercellular adhesive properties, ultimately leading to cell aggregation and subsequent development of a multicellular, multilayered biofilm architecture (Costerton et al., 1995). Parallel to the discovery of factors with dedicated functions during primary attachment, the nature of intercellular adhesins, functioning as the “biofilm glue,” was partially unraveled. Based on the early electron microscopic studies, showing *S. epidermidis* cells embedded in an amorphous extracellular matrix (Peters et al., 1981), focus was set onto the biochemical analysis of biofilm matrix components. These efforts ultimately resulted in the discovery of the polysaccharide intercellular adhesin (PIA), which is at present the most extensively studied intercellular adhesin (Mack et al., 1994b).

Structural analysis of PIA and comparison of PIA isolated from *S. epidermidis* and *S. aureus* has been recently reviewed (Mack et al., 2013). The structure of PIA was first described for biofilm-forming *S. epidermidis* 1457 and RP62A. PIA was extracted from the cells by sonication after the strains had been cultured in trypticase soy broth, which revealed the existence of both a major polysaccharide I (>80%), and a minor polysaccharide II (<20%), which are structurally closely related and could be separated due to differing ionic properties (Mack et al., 1996). Chemical analyses and NMR spectroscopy have demonstrated that polysaccharide I is a linear homoglycan of β-1,6-linked 2-amino-2-deoxy-D-glucopyranosyl residues. Approximately 80–85% of them are *N*-acetylated; the rest are non-*N*-acetylated and carry a positive charge. Polysaccharide II of PIA has a lower proportion of de-N-acetylated 2-amino-2-deoxy-D-glucopyranosyl residues and is modified by ester-linked succinate residues rendering it anionic (Mack et al., 1996). Despite a high apparent molecular weight indicated by elution in the void volume of Sephadex G200 (Mack et al., 1994a, 1996) or Sephacryl S300 columns (C. Fischer and D. Mack, unpublished results), the ratio of reducing terminal sugar residues to total sugar residues was shown by methylation analysis to be 1:130, implying an average M_r_ of 30,000 for PIA polysaccharide chains (Mack et al., 1996). This implies aggregation of PIA polysaccharide chains in solution. PIA was shown to function also as the hemagglutinin of *S. epidermidis* (Rupp and Archer, 1992; Fey et al., 1999; Mack et al., 1999). Production of a functionally active PIA molecule requires expression of all four *icaADBC* genes (Gerke et al., 1998). The process has been the subject of detailed study in recombinant strains of *S. carnosus* which expressed different combinations of the *icaADBC* genes and with UDP-GlcNAc as a sugar donor (Gerke et al., 1998). IcaA belongs to the glycosyltransferase 2 family. It is an integral membrane protein with 412 aa and four predicted transmembrane domains (Heilmann et al., 1996; Gerke et al., 1998; Gill et al., 2005), and directs the synthesis of β-1,6-linked GlcNAc oligosaccharides of up to 20 GlcNAc units. IcaD is required for full activity of IcaA *in vitro*. It is a 101 aa integral membrane protein with two potential membrane spanning domains: it may be a chaperone directing folding and membrane insertion of IcaA and may act as a link between IcaA and IcaC (Gerke et al., 1998). Also essential for the synthesis of fully functioning PIA is IcaC, a 355 aa integral membrane protein with 10 predicted transmembrane domains, which may be involved in externalization and elongation of the growing polysaccharide (Gerke et al., 1998). IcaB is a member of the polysaccharide deacetylase family, including, for example, chitin deacetylases or the chitooligosaccharide deacetylase NodB of *Rhizobium melioti*. In its mature form it is a 259 aa secreted protein with a predicted signal sequence, responsible for the de-*N*-acetylation of PIA, and crucial for PIA activity in biofilm formation and for virulence in *S. epidermidis* (Vuong et al., 2004a). In Δ*icaB*-mutants, where the *icaB* gene has been deleted, PIA is poorly retained on the cell surface as it does not contain non-*N*-acetylated GlcNAc (Vuong et al., 2004a).

Early observations made by biochemical analysis of biofilm matrix extracts not only showed the presence of(poly-)saccharides but pointed toward the additional presence of proteins and nucleic acids (Hussain et al., 1991). Specific proteins have been identified and characterized. Apart from biofilm associated protein Bap (Tormo et al., 2005a), that is only rarely found in invasive *S. epidermidis* from human infections (Tormo et al., 2005a; Rohde et al., 2007; Piessens et al., 2012), SesC has been proposed to play a role in biofilm formation (Shahrooei et al., 2009). SesC is an LPXTG motif-containing 68 kDa surface protein of *S. epidermidis* distantly related to clumping factor A of *S. aureus* and is expressed more strongly in biofilm-associated as compared to planktonic *S. epidermidis* 1457 and 10b cells *in vitro* and *in vivo* (Shahrooei et al., 2009; Lam et al., 2014). Rabbit anti-SesC inhibited biofilm formation of a number of *S. epidermidis* isolates *in vitro*, which may be related to changes in primary attachment to fibrinogen-coated surfaces in the presence of anti-SesC. All of 105 *S. epidermidis* isolates recovered from nose swabs or infections were in possession of the *sesC* (Shahrooei et al., 2009). Passive and active immunization using SesC as a target protein was shown to decrease *S. epidermidis* biofilm formation in an *in vivo* model of central venous catheter infections (Shahrooei et al., 2012). A specific role of SesC as an intercellular adhesin in biofilm accumulation remains to be demonstrated.

## Multifunctional protein factors in *S. epidermidis* biofilm formation

Although having partially additional enzymatic functions (e.g., AltE, GehD), it is a common feature of many factors contributing to *S. epidermidis* surface colonization that they carry out functions either during the primary attachment or accumulative phase of biofilm formation (Rohde et al., 2006; Mack et al., 2009; Otto, 2009). With the increasing interest in protein factors contributing to staphylococcal biofilm accumulation, it became apparent that, at least in *S. aureus*, many factors (e.g., FnBPA, ClfA) must be regarded as multifunctional proteins not having an exclusive role in either primary attachment or accumulation (Foster et al., 2014). This concept of multifunctional proteins with important roles during several phases of biofilm formation and surface colonization is now also evolving in *S. epidermidis*, with the accumulation associated protein (Aap) and the extracellular matrix binding protein (Embp) being the most prominent factors.

**Figure 1:**
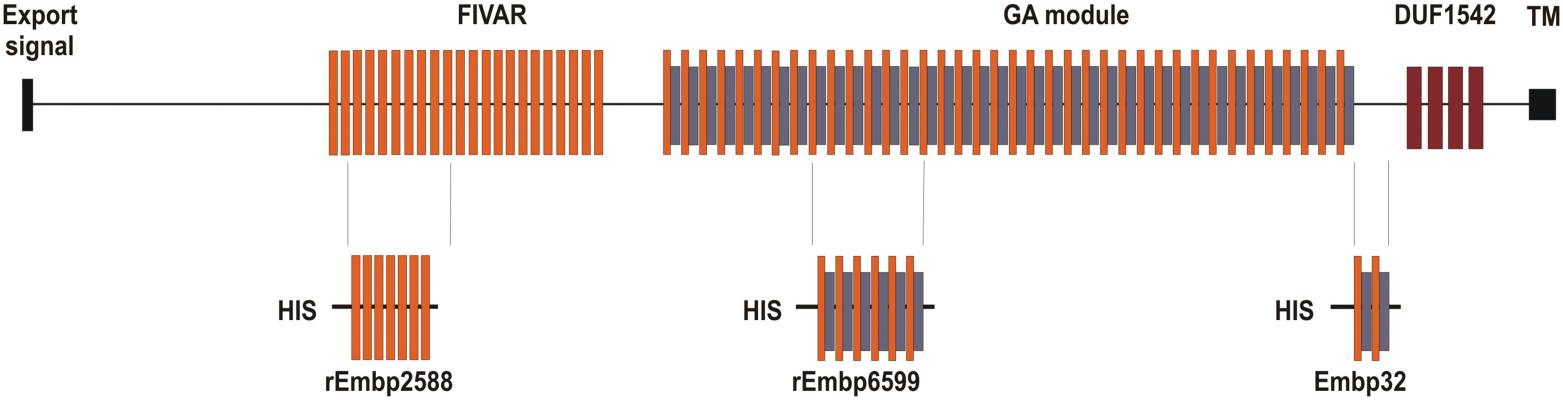
**Schematic representation of Embp and relative position of recombinant proteins for which functional data are available**. Embp possesses an N-terminal YSIRK motif containing export signal, which is followed by a region of unordered conformation. The central part is comprised of the FIVAR or FIVAR-GA repeats, while the C-terminal end comprises up by four Domains of Unknown Function (DUF) and a putative transmembrane (TM) region (Christner et al., 2010). Embp32 is a recombinant Embp fragment (aa 9180–9421) from *S. epidermidis* NCTC11047 spanning four alternating FIVAR and FIVAR-GA repeats and that exhibits fibronectin binding capacity (Williams et al., 2002). rEmbp2588 (aa 2588–3187) contains seven FIVAR-only repeat units, while rEmbp6599 (aa 6599–7340) consists of six G-related albumin binding (GA) modules intercalated by FIVAR repeats. The rEmbp2588 and rEmbp6599 both bind to surface immobilized fibronectin (Christner et al., 2010).

Embp and its ortholog in *S. aureus* designated Ebh were almost simultaneously identified during studies aiming at identifying *S. epidermidis* or *S. aureus* protein factors with Fn binding activities (Clarke et al., 2002; Williams et al., 2002). By using a phage display approach a phage was isolated, which exhibited fibronectin binding activity and contained a DNA fragment from a 30,500 bp open reading frame (ORF) coding for a 10,203 aa protein that was referred to as extracellular matrix binding protein Embp (Williams et al., 2002) (Figure 1). Using bioinformatics the architecture of Embp was predicted to consist of an N-terminal YSIRK-motif containing export signal (aa 58–84), followed by an unordered region of approximately 2500 amino acids (aa 85–2586). The overall architecture of Embp mainly is characterized by 21 repetitive “Found In Various Architectures” (FIVAR) repeats (aa 2587–4500) and 38 alternating “G-related Albumine-binding” (GA) motifs and FIVAR repeats—termed the FIVAR-GA repeats—(aa 4501–9443) that span roughly 7000 amino acids in the central proportion of the Embp protein. Finally the C-terminus consists of four domains of unknown function (DUF1542) (aa 9444–9841), followed by a potential transmembrane motive (aa 10,070–10,088) (Christner et al., 2010) (Figure 1). Although the *S. aureus* homolog Ebh displays at least functional homologies with respect to fibronectin binding activity (Clarke et al., 2002) some functional predictions found in Ebh, e.g., an N-terminal hyperosmolarity resistence domain (Kuroda et al., 2008; Tanaka et al., 2008) have not been identified in the Embp. The overall structural organization of the Ebh protein seems to be more or less identical to Embp, but in detail gradual differences are apparent, e.g., only seven FIVAR motifs but 12 additional FIVAR-GA repeats and four additional DUF1542 repeats compared to Embp were predicted (Tanaka et al., 2008). In fact, it appears that among different *S. aureus* strains the number of repetitive modules (FIVAR and FIVAR-GA) within Ebh is variable, while available sequence data shows no variability of these features in Embp from *S. epidermidis* RP62A, ATCC12228 or 1585.

Crystallization of two 126 amino acid FIVAR-GA repeats from EbhA of *S. aureus* MU50 (termed EbhA-R7-R8) revealed a triple α-helical structure interconnected by a continuous alpha helical string displaying an elongated shape (Sakamoto et al., 2008; Tanaka et al., 2008). In fact, the corresponding FIVAR-GA repeat of Embp exhibits a very similar, if not identical structure (Büttner, Perbandt, Rohde, unpublished results). Additional preliminary structural analysis of repetitive FIVAR regions (Büttner, Perband, Rohde, unpublished results) or DUF1542 repeats (Linke et al., 2012) suggest that overall Embp constitutes an elongated rod-like conformation.

In collections of clinical significant *S. epidermidis* isolates *embp* was detected in more than 90% of strains (Rohde et al., 2004, 2007). In addition, evidence for the expression of Embp *in vivo* resulted from investigations showing the presence of anti-Embp antibodies in patients with confirmed *S. epidermidis* prosthetic joint infections (Mack, Büttner, Rohde, unpublished). Strikingly, using a flow cell model of biofilm formation, anti-Embp antibodies were shown to inhibit *S. epidermidis* 1457 biofilm formation (Lam et al., 2014), making Embp a potential candidate for preventive strategies (Götz, 2004).

Experimental evidence primarily suggested a role of Embp in primary attachment. Importantly, over-expression of Embp did not alter binding to unmodified polystyrene, but only boosted bacterial adherence to Fn-coated surfaces (Christner et al., 2010), and Embp–Fn interactions were necessary for biofilm accumulation on plastic surfaces that otherwise did not promote bacterial binding (Christner et al., 2010). Results from phage display suggested that FIVAR-GA repeats were relevant to the Fn-binding activity of Embp (Clarke et al., 2002; Williams et al., 2002). This assumption was later validated by biochemical analysis showing direct evidence for interactions between a recombinant protein containing FIVAR-GA repeats (Christner et al., 2010). In addition, these studies also found evidence that FIVAR-modules alone are capable of binding to immobilized Fn (Christner et al., 2010) (Figure 1). The vast majority of bacterial Fn-binding proteins bind to Fn via interactions with the N-terminal Fn type I domains (Bingham et al., 2008; Chagnot et al., 2012). This is especially true for the *S. aureus* Fn binding protein FnBPA (Meenan et al., 2007). Although the exact mechanism of Embp–Fn interaction awaits definitive molecular analysis, it is already clear that Embp uses an mechanism for Fn interactions independent of type I Fn modules, but involving Fn type III modules located at the C-terminus of Fn (Bustanji et al., 2003), most likely FnIII_12−14_ (Christner et al., 2010). This type of interaction has only rarely been described in bacterial pathogens (Kingsley et al., 2004; Dabo et al., 2006).

In addition to its function in primary attachment, Embp is also functional as an intercellular adhesin. The intercellular adhesive properties and biofilm inducing activity of Embp was first detected in a laboratory derived strain *S. epidermidis* 1585v that, by a spontaneous chromosomal rearrangement, overexpressed a truncated isoform of Embp referred to as Embp1 (Christner et al., 2010). A transposon insertion within Embp1 resulted in abolished biofilm formation. By placing an inducible promoter in front of the wild-type *embp*, a biofilm inducing effect of full length Embp became apparent (Christner et al., 2010), proving the intercellular adhesive properties of Embp. Of notice, up-regulation of *embp* is also associated with resistance against up-take by professional phagocytes (Schommer et al., 2011). Studies on the overall impact of Embp on *S. epidermidis* cell wall assembly and its relation to immune-escape will shed light on the question if in this species, the giant protein carries similar functions as compared to Ebh in *S. aureus* (Cheng et al., 2014).

While at the time of its identification Embp appeared primarily as a factor mediating primary attachment, Aap was initially, and as already suggested by its designation, thought to confer intercellular adhesion and thereby to contribute to biofilm accumulation. Aap is a covalently linked, cell wall associated protein consisting of an A- and a B-domain (Rohde et al., 2005; Gruszka et al., 2012; Conrady et al., 2013; Schaeffer et al., 2015). The 584 aa A domain harbors a N-terminal export signal, several imperfect, 16 amino acid repeats, and a globular 212 amino acid region with predicted α-helical and β-sheet content. The 212 amino acid region is highly conserved between Aap and its *S. aureus* ortholog SasG, and bioinformatical analysis predicts that this domain possesses lectin-like activity (Schaeffer et al., 2015). The B domain consists of a varying number of repetitive 128 amino acid repeats (Rohde et al., 2007). Variations of B repeats not only exist between independent *S. epidermidis* strains (e.g., reference strain RP62A possess 13 repeats, while *S. epidermidis* 1457 only harbors seven repeats) (Monk and Archer, 2007; Schaeffer et al., 2015), but are also encountered in clonally identical clinical isolates subsequently recovered during the course of device infections from individual patients (Rohde et al., 2007). This observation lead to the hypothesis that Aap B repeat variations could represent a mechanism contributing to *S. epidermidis* immune escape through modification of major cell surface epitopes (Rohde et al., 2007).

Aap can be detected on the bacterial cell wall, where it is most likely retained by covalent linkage to the peptidoglycan via its C-terminal gram-positive anchor region (Hussain et al., 1997; Rohde et al., 2005; Schommer et al., 2011; Conlon et al., 2014). A more detailed analysis using confocal microscopy demonstrated that within living, three dimensional *S. epidermidis* biofilms, Aap strictly localizes to the bacterial cell surface, while only minimal amounts are released into the biofilm matrix itself (Schommer et al., 2011). These results are underpinned and extended by electron-microscopic studies showing that Aap forms elongated fibers that project 120 nm away from the cell wall in localized tufts (Banner et al., 2007). Recently, using a structural biology approach, the molecular basis of this intriguing spatial organization was determined. An X-ray crystallography derived high resolution model of different recombinant proteins from the B region of *S. epidermidis* Aap (Conrady et al., 2013) or *S. aureus* SasG (Gruszka et al., 2012) showed that each B repeat consists of two regions, an approximately 80 aa G5 domain and an approximately 50 aa linker region referred to as E-region that interconnects repetitive G5 domains (Gruszka et al., 2012). The G5 domains each comprise two successive three-stranded β-sheets connected by triple-helix-like regions, while the E region is composed of two β-sheets (Gruszka et al., 2012; Conrady et al., 2013). E sequences fold cooperatively and form interlocking interfaces with G5 domains in a head-to-tail fashion, resulting in a contiguous, elongated, monomeric structure. Although E and G5 domains lack a compact hydrophobic core, G5 domain and multidomain constructs thereof have thermodynamic stabilities only slightly lower than globular proteins of similar size, explaining why Aap could form protruding fibers even under harsh environmental conditions (Gruszka et al., 2012; Conrady et al., 2013).

The functional importance of Aap for *S. epidermidis* biofilm formation was first recognized during studies in which chemically derived, biofilm-negative mutant M7 of *S. epidermidis* RP62A was analyzed (Hussain et al., 1997). Mutant M7 failed to express Aap on the cell surface, and antibodies raised against Aap were able to inhibit biofilm formation in biofilm-positive parent strain RP62A (Hussain et al., 1997). Later, Aap was independently picked up in experiments in which cell surface proteins of a clinically significant but biofilm-negative *S. epidermidis* wild-type strain 5179 were compared with those isolated from a laboratory derived, biofilm-positive revertant of that strain, referred to as 5179-R1. In protein preparations from 5179-R1 reduced amounts of full length Aap were detected, while in parallel, a shorter, roughly 140 kDa Aap isoform became apparent (Rohde et al., 2005). By using mass spectrometry and N-terminal sequencing evidence was created that the 140 kDa isoform mainly consists of repetitive B domain, mapping to aa 596 of the mature Aap protein. Rabbit antiserum raised against recombinantly expressed B domain inhibited biofilm formation by strain 5179-R1, not only directly supporting the idea of a functional involvement of Aap, but moreover indicating the crucial importance of B domain during this process. Indeed, genetic studies corroborated this hypothesis, showing that *in trans* expression of B domain in biofilm-negative *S. epidermidis* 1585 and surrogate host *S. carnosus* TM300 was sufficient to induce a biofilm-positive phenotype (Rohde et al., 2005). Importantly, expression of B domain did not alter the primary adherence properties, but resulted in cell aggregation, showing that in fact, Aap can be regarded as an intercellular adhesin (Rohde et al., 2005; Conrady et al., 2008; Geoghegan et al., 2010). Indeed, the importance of B domain for intercellular adhesion was also described for SasG in an *S. aureus* background (Geoghegan et al., 2010). The intercellular adhesive properties can be partially explained by Zinc-dependent homodimerization of B domains (Conrady et al., 2008, 2013), however, some evidence suggests the existence of additional, heterotypic interactions involved in Aap B domain mediated biofilm accumulation (Decker et al., unpublished results).

Importantly, expression of full length mature Aap is not sufficient to mediate intercellular adhesion during the second, accumulative phase of biofilm formation, but to become functionally active as an intercellular adhesin, Aap requires proteolytic processing, resulting in the removal of the A domain (Rohde et al., 2005; Geoghegan et al., 2010). Since Aap processing does not regularly occur under *in vitro* growth conditions (Rohde et al., 2005; Schaeffer et al., 2015), this phenomenon is a reasonable explanation for the finding that Aap-expressing *S. epidermidis* not necessarily form a biofilm (Rohde et al., 2007).

Although the intercellular adhesive Aap properties were recognized first, there is now mounting evidence supporting a significant role of Aap also in primary attachment to natural epithelial cells or artificial surfaces (Macintosh et al., 2009; Conlon et al., 2014; Schaeffer et al., 2015). Intriguingly, early work already pointed toward a role of domain A in this process, in which Aap B domain most likely is of only minor functional importance (Macintosh et al., 2009). Binding of Aap expressing *S. epidermidis* NCTC 11047 to squamous epithelial cells was partially inhibited by the addition of recombinant Aap A domain, as was binding of several additional, Aap-positive clinical *S. epidermidis* strains (Macintosh et al., 2009). Moreover, *in trans* expression of A domain in surrogate host *L. lactis* enabled the bacteria to more efficiently adhere to corneocytes as compared to a *L. lactis* strain that expressed B domain alone (Macintosh et al., 2009), thus providing genetic evidence for a potential role of A domain in colonization of natural skin surfaces. In extension to the work of Macintosh and co-workers, the role of Aap A domain in primary attachment was further refined in two studies addressing the question as to which extent the A domain could also contribute to colonization of abiotic surfaces (Conlon et al., 2014; Schaeffer et al., 2015). In a clinical *S. epidermidis* isolate CSF41498, expressing an unprocessed mature, i.e., A domain containing Aap on the surface, attachment to polystyrene was almost completely abolished after deletion of *aap*. In addition, attachment was significantly inhibited by an antiserum raised against Aap A domain, whereas anti-Aap domain B antiserum had no significant impact on adherence capacities of that strain (Conlon et al., 2014). Intriguingly, deletion of *aap* in *S. epidermidis* 1457, expressing a processed Aap isoform devoid of the A domain, did not render the adherence capacities of that strain (Schaeffer et al., 2015). Moreover, anti-Aap A domain antiserum had no effect on binding to the surface of *S. epidermidis* 1457, supporting the idea that indeed, the presence of A domain, i.e., expression of a mature unprocessed Aap, is essential for Aap-mediated surface adherence. Indeed, *in trans* expression of full length Aap in 1457Δ*aap* resulted in improved bacterial binding (Schaeffer et al., 2015). Direct genetic evidence for an involvement of Aap A domain in staphylococcal plastic adherence is demonstrated by *in trans* expression of A domain fused to the C-terminal cell wall anchor in *S. carnosus* TM300, which significantly improved bacterial binding as compared to the wild-type strain or a strain expressing the Aap B domain (Schaeffer et al., 2015). Thus, a new picture of Aap evolved in which the protein plays a bifunctional role in both, the very early primary attachment phase as well as the later accumulative phases of biofilm formation, and that Aap's inherent functionalities are represented by separated and structurally distinct domains within the protein (Rohde et al., 2005; Macintosh et al., 2009; Conlon et al., 2014; Schaeffer et al., 2015).

## Molecular interactions and regulatory events during *S. epidermidis* biofilm formation

The detailed analysis of functional molecules contributing to *S. epidermidis* biofilm formation has clearly revealed that primary attachment as well as biofilm accumulation is mediated by redundantly organized factors with remarkable exclusive properties, but certainly broadly overlapping functional characteristics. The latter aspect is especially true for molecules active during the accumulative phase: production of PIA, Aap or Embp all efficiently induce cell aggregation, ultimately leading to assembly of a biofilm consortium. However, given the common observation that in clinical *S. epidermidis* isolates genes encoding for the PIA-synthesis machinery (*icaADBC*), *aap, embp*, and additional adherence-associated factors are all widely distributed, with a large percentage of strains carrying all three genes (Frebourg et al., 2000; Galdbart et al., 2000; Klug et al., 2003; Arciola et al., 2004; Rohde et al., 2004, 2007; Petrelli et al., 2006), the question is put forward if and how these mechanisms interact, and which consequences co-expression of distinct intercellular adhesins might have for *S. epidermidis* biofilm accumulation.

Indeed, the idea that distinct *S. epidermidis* intercellular adhesins cooperate during biofilm assembly is supported by epidemiological work identifying that strains being positive for *icaADBC* and *aap* appear to form stronger biofilms compared to strains being positive only for *icaADBC* or *aap* (Stevens et al., 2008). Bioinformatic analysis of *aap* and *embp* indeed suggests that direct interaction with PIA might be possible. The G5 domains of the Aap B domain are postulated to have N-acetylglucosamine binding activity (Bateman et al., 2005), and similarly, FIVAR regions of Embp are likewise predicted to have sugar binding potential (Christner et al., 2010), making the direct interaction between proteinacous intercellular adhesins and N-acetylglucosamine containing PIA possible. However, so far no experimental data are available that would support this important and interesting hypothesis. At present, it rather appears that parallel expression of specific intercellular adhesins introduces functional redundancy into biofilm accumulation, i.e., the intercellular adhesive properties of distinct adhesins functionally substitute for each other. Experiments, in which the susceptibility of biofilms of Embp-producing *S. epidermidis* strain 1585v against treatment with proteases was tested, revealed that, while being naturally sensitive against protease activity, additional *in trans* expression of *icaADBC* and PIA production protected the *S. epidermidis* 1585v biofilm from proteolytic breakdown (Christner et al., 2010). On the other hand, while PIA-dependent biofilms are readily disrupted by PIA-degrading enzyme DspB (Kaplan et al., 2004; Rohde et al., 2007), parallel expression of a proteinacous intercellular adhesin Embp and PIA rescued a biofilm-positive phenotype even in the presence of DspB (Christner et al., 2010). Functional substitution is also evident for Aap and PIA: the inactivation of *aap* in a PIA-producing genetic background had no apparent effect on biofilm formation in *S. epidermidis* 1457 as tested by conventional crystal violet biofilm assays or confocal laser scanning microscopy, probably because biofilm formation is already maximal in the various assay systems when PIA is expressed alone (Schaeffer et al., 2015). On the other hand, *in trans* expression of Aap B domain, being sufficient for induction of cell aggregation, induces biofilm formation in a PIA-negative, *icaADBC* mutant 1457-M10 (Henke and Rohde, unpublished results).

The interpretation of functional redundancy in intercellular adhesive molecules as a simple means allowing *S. epidermidis* to form as robust biofilms as possible, however, might be an inappropriate oversimplification of their actual functional importance during different *S. epidermidis* live styles, ranging between colonization and (foreign-material associated) host invasion. In that respect, it is important to acknowledge that a more detailed morphological analysis of PIA-, Aap- or Embp dependent biofilms revealed that these biofilm types differ significantly in their morphological properties. While in PIA-dependent biofilms, *S. epidermidis* cells are embedded into meshwork of PIA-containing extracellular matrix fibers, Aap production induces formation of densely packed cell layers that evenly cover the surface. In Embp-dependent biofilms, bacteria produce small amounts of Embp-containing extracellular matrix structures, however, these biofilms differ from PIA-dependent cell consortia by the lack of towers and clusters (Schommer et al., 2011). Overall, PIA-dependent biofilms are significantly more stable against washing procedures as compared to protein-dependent biofilms, indicating their inherent, pronounced mechanical robustness. Thus, the specific biological properties of a given intercellular adhesin could constitute a way how *S. epidermidis* can cope with varying challenges during colonization and infection (Schommer et al., 2011). Analysis of invasive *S. epidermidis* strains from various types of infections supports the idea that in fact, the ability to differentially make use of specific intercellular adhesins equips *S. epidermidis* to specifically adapt to changing environments with potential fundamentally different requirements, e.g., presence of mechanical or osmotic stress, or exposure to effectors of the host immune system (Otto, 2014). Specifically, *S. epidermidis* strains from central venous catheter infections, i.e., a situation with significant exposure to mechanical stress and cellular and soluble factors of innate immunity (e.g., complement factors) are more likely to carry *icaADBC* compared to strains from prosthetic joint infections, i.e., an infection setting characterized by static conditions at the implant–tissue interface (Ziebuhr et al., 1997; Arciola et al., 2004; Rohde et al., 2004, 2007; Stevens et al., 2008; Mack et al., 2013).

Additional evidence suggesting that usage of specific intercellular adhesins indeed follows an adaptive program results from the observation that PIA-dependent biofilm formation on one hand, and Aap- and Embp-dependent biofilm formation on the other, are, at least under *in vitro* growth conditions, mutually exclusive (Rohde et al., 2005, 2007; Christner et al., 2010). In fact, under standard growth conditions in trypticase soy broth (TSB), neither Aap nor Embp-dependent biofilm formation would have been identified, since here Aap is not properly processed, while *embp* is not expressed at all (Rohde et al., 2005; Christner et al., 2010). The characterization of these intercellular adhesins only was possible by analysis of laboratory derived, spontaneous mutants or by introduction of artificial promoters allowing for inducible gene expression, respectively (Christner et al., 2010; Rohde et al., 2005). Thus, PIA- and Aap- or Embp-dependent modes of biofilm formation are obviously under the control of opposed regulatory circuits. For PIA-dependent biofilm formation, a very detailed picture of how *icaADBC* expression is integrated into a complex superimposed regulatory network has evolved. Others have recently reviewed this field in detail (Mack et al., 2004; Cue et al., 2012). In brief, several regulators of *icaADBC* expression and PIA synthesis were identified (Xu et al., 2006; Sadykov et al., 2011; Wang et al., 2011b), with sigma factor B and staphylococcal accessory regulator SarA being the most prominent (Fluckiger et al., 1998; Knobloch et al., 2001, 2004; Tormo et al., 2005b; Handke et al., 2007). Especially, negative regulator of *icaADBC* expression, IcaR, has been elucidated in great detail (Conlon et al., 2002). However, the regulation of PIA-independent mechanism of *S. epidermidis* biofilm formation remained obscure.

More recently, in an attempt to shed light onto potential negative regulators interfering with biofilm formation in clinical *S. epidermidis* isolates under *in vitro* conditions, a transposon mutant library established in biofilm-negative *S. epidermidis* 1585 was screened for biofilm-positive mutants (Christner et al., 2012). Independent biofilm-positive mutants were identified, and further analysis showed that all carried Tn*917* insertions in *sarA*. Intriguingly, inactivation of *sarA* induced a strong up-regulation of *embp* expression, and additional experimental work proved that Embp was necessary for 1585Δ*sarA* biofilm formation. Moreover, higher eDNA amounts were present in biofilms of 1585Δ*sarA*, and this finding was related to increased autolysis, itself being a result of over-production of metalloprotease SepA (Lai et al., 2007) and subsequent processing and functional activation of AtlE (Christner et al., 2012). Of notice, protease-mediated autolysin processing, augmented autolysis and subsequent eDNA release and biofilm formation has also been described in *Enterococcus faecalis* as part of a fratricidal mechanism (Thomas et al., 2008, 2009).

**Figure 2:**
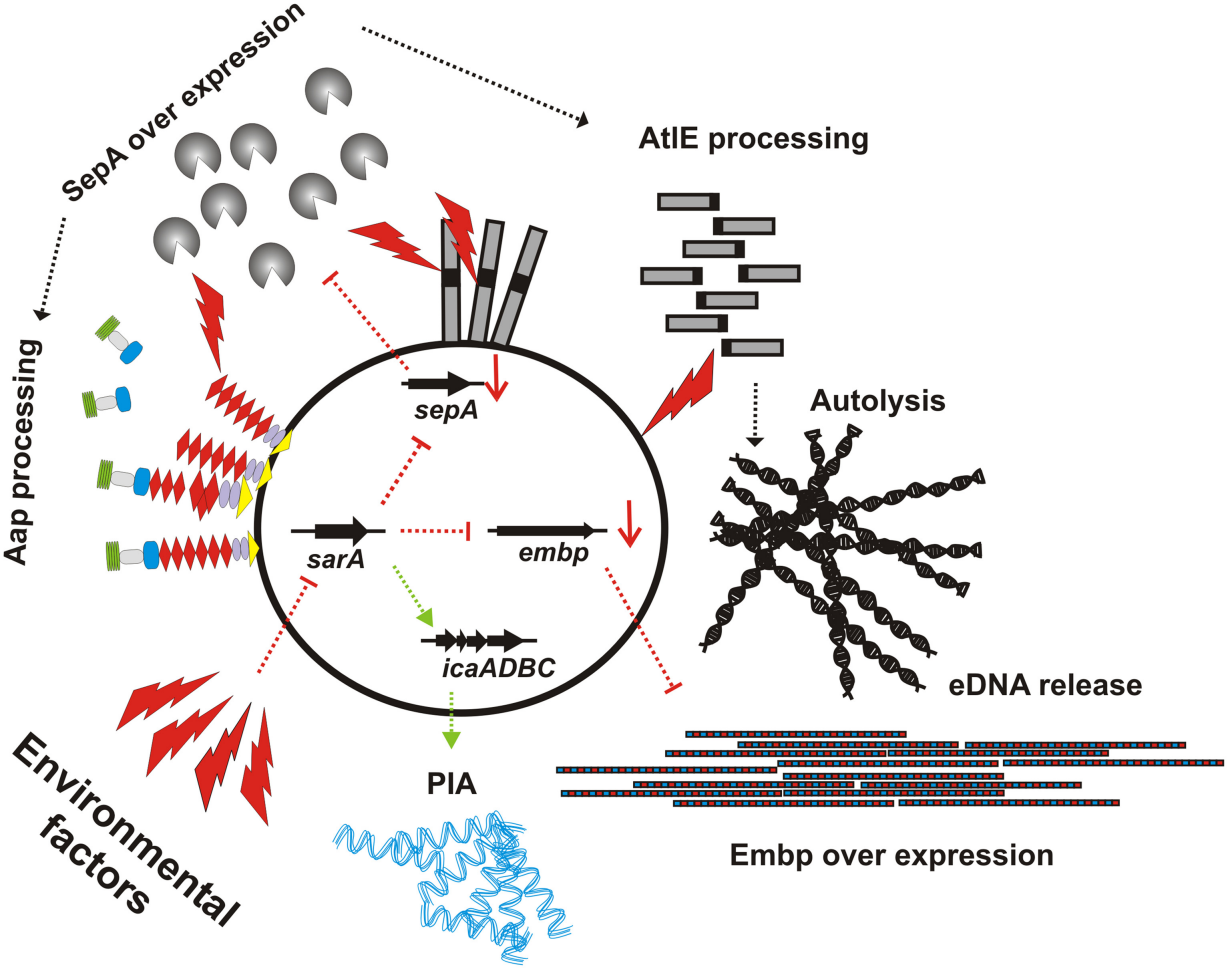
**Schematic representation of SarA effects on expression of independent intercellular adhesins**. While in trypticase soy broth, *sarA* is expressed, leading to *icaADBC* expression and PIA dependent biofilm formation, down-regulation of *sarA* leads to an de-repression of *embp* expression, allowing for maintenance of a biofilm-positive phenotype despite *icaADBC* down-regulation and loss of PIA production. Parallel to *embp*, following inactivation of *sarA*, metalloprotease *sepA* is up-regulated, leading to increased AtlE processing, autolysis and subsequent eDNA release. Potentially, the over-production of SepA also contributes to Aap-dependent biofilm accumulation by proteolytically processing of the mature protein, resulting in the removal of it's A domain. Environmental (potentially also host) factors that repress *sarA* expression are unknown, as are the pathways via which *sarA* itself is regulated.

The results of the study by Christner and co-workers appear to contradict previous findings, showing that SarA is a positive regulator of *S. epidermidis* biofilm formation by augmenting *icaADBC* expression (Tormo et al., 2005b; Handke et al., 2007). However, a more detailed analysis of SarA function in PIA-positive background of *S. epidermidis* 1457 showed that in this strain, inactivation of *sarA* does not completely abolish biofilm formation (Handke et al., 2007). Even inactivation of *icaADBC* in 1457Δ*sarA* did not render the strain biofilm-negative, and in fact, the biofilm of 1457-M10Δ*sarA* is Embp- and eDNA-dependent (Christner et al., 2012). In conclusion, the study by Christner and co-workers establishes a key role for SarA in controlling the mode of biofilm formation in *S. epidermidis*: up-regulation of *sarA* shifts *S. epidermidis* toward production of a PIA-dependent type of biofilm, whereas down-regulation of *sarA* supports formation of PIA-independent types of biofilm formation (Figure 2). Clearly, the regulation involves transcriptional effects with direct consequences for intercellular aggregation (i.e., *embp* and *icaADBC* up- or down-regulation), but also post-translational mechanisms of regulation exerted via up- or down-regulation of metalloprotease SepA with subsequent proteolytic processing and functional modification of cell surface proteins (e.g., AtlE). Importantly, through the latter mode of action SarA could also influence Aap-dependent biofilm formation by boosting proteolytic removal and functional activation of Aap B-domain (Figure 2). In the future it will be of major interest as to which extent the SarA regulatory circuit and interrelated additional regulators, e.g., *agr* (Vuong et al., 2004b), *ssrAB* (Wu et al., 2015), *saeRS* (Lou et al., 2011), or *codY* (Batzilla et al., 2006) as well as the levels of proteolytic activity influence the balanced formation of PIA-dependent or -independent types of biofilm formation.

Certainly, the highly artificial character of studies related to the function of SarA makes it difficult to draw general conclusions on the relevance of this mechanism during *in vivo* biofilm accumulation. In that respect, it becomes clearer that that more appropriate models of biofilm formation are generally needed in order to reflect the *in vivo* situation during infection and colonization. These models must take into account the potential inappropriateness of TSB as a growth medium that obviously introduces a significant bias toward PIA-dependent biofilm formation into the experimental system. The finding that *embp*, but also additional factors involved in *S. epidermidis* biofilm formation (e.g., Fbe/SdrG) are only expressed in the presence of serum (Sellman et al., 2005, 2008; Christner et al., 2010), clearly argues for experimental set-ups that mimic environmental conditions in ecological niches usually encountered by *S. epidermidis*, e.g., within the vestibulum nasi (Krismer et al., 2014) or on the skin (Ohnemus et al., 2007; Olson et al., 2014).

## Relevance of *S. epidermidis* biofilm formation for the pathogenesis of foreign-material associated infections *in vivo*

In general, studies aiming at elucidating the clinical relevance of biofilm formation and the relative contribution of specific factors to foreign-material colonization and establishment of a chronic persistent infection are either studied using cell culture models (Vuong et al., 2004a,c; Schommer et al., 2011), *Caenorhabditis elegans* (Begun et al., 2007) or animal models of device infections, e.g., central venous catheter or prosthetic device infection models (Wang et al., 2011a; Odekerken et al., 2013; Scherr et al., 2014b; Schaeffer et al., 2015). In early studies on the importance of biofilm formation *in vivo* using animal models and genetically unrelated, biofilm-positive or biofilm-negative *S. epidermidis* isolates failed to demonstrate conclusive evidence that biofilm forming isolates are more virulent compared to biofilm-negative strains. However, subsequent studies using genetically defined, isogenic pairs of biofilm-positive wild-type and biofilm-negative mutants were more conclusive. In a subcutaneous catheter infection model in mice and a central venous catheter infection model in rats biofilm-positive, PIA-producing *S. epidermidis* 1457 was more virulent than its isogenic biofilm-negative transductant 1457-M10 (Rupp et al., 1999a,b). An AtlE-deletion mutant of this strain was also attenuated (Rupp et al., 2001). In a rat central venous catheter model, expression of *icaRADBC* in *icaADBC*-negative *S. epidermidis* strains also led to increased virulence (Li et al., 2005). A *Caenorhabditis elegans* infection model was used to study biofilm-positive *S. epidermidis* 9142, showing attenuation of an *icaA* insertion mutant compared to its isogenic, PIA producing parent strain 9142 (Begun et al., 2007). Virulence was restored to wild-type in the biofilm-negative mutant by complementation with cloned *icaADBC* (Begun et al., 2007). However, in a collection of *S. epidermidis* infective endocarditis isolates, PIA expression and pathogenicity for *C. elegans* was not closely associated (Monk et al., 2008). More recently, using a catheter infection model in which realistic colonization modalities were chosen (i.e., infection after catheter insertion and not usage of pre-colonized materials) the inactivation of *icaADBC* had no apparent effect on colonization, while *aap* inactivation almost completely abolished the ability of *S. epidermidis* to establish an infection (Schaeffer et al., 2015).

A significant reason for the impaired pathogenicity of *icaADBC*-negative mutants in animals models is the improved ability of the innate immune system to clear biofilm-negative *S. epidermidis* (Schommer et al., 2011). There is significant experimental evidence from cell culture assays that indeed biofilm positive strain 1457 was less susceptible to killing by antimicrobial peptides and also displayed decreased phagocytosis and killing by polymorphonuclear granulocytes (PMNs) compared to its isogenic *icaA* mutant 1457-M10 (Vuong et al., 2004c). When *S. epidermidis* 1457 was either grown in a static biofilm or planktonic culture, the organism grown in a biofilm was less susceptible to phagocytic killing after opsonisation with normal human serum, as was an isogenic biofilm-negative *icaA*-insertion mutant (Kristian et al., 2008). PIA-dependent biofilm formation also interferes with host complement activation. Biofilm-positive wild-type bacteria pre-opsonised with normal human serum were more resistant to complement-dependent killing than the corresponding isogenic biofilm-negative bacteria (Kristian et al., 2008). There is, moreover, evidence that *S. epidermidis* biofilm formation interferes with phagocytic up-take and with pro-inflammatory activation of macrophages. This effect was irrespective of the intercellular adhesin used (Schommer et al., 2011). These phenotypes clearly could additionally contribute to the chronic persistent, low-grade inflammatory course of a *S. epidermidis* infection.

It is important to stress that, since *S. epidermidis* is an opportunistic pathogen, mechanisms of pathogenicity which are important in some types of device-related infection might be less crucial in others. For example, in the guinea pig tissue cage model (Zimmerli et al., 1982) there was no difference in virulence between a biofilm-positive wild-type *S. epidermidis* 1457 and its isogenic *icaA*-insertion mutant, and no difference between *icaADBC*-positive and -negative clinical isolates (Francois et al., 2003; Chokr et al., 2007). Nonetheless PIA was expressed *in vivo* in the tissue cages, and when animals were infected with both strains at the same time, the wild-type out-competed the mutant (Fluckiger et al., 2005). This may be because phagocytes are severely impaired in tissue cages (Zimmerli et al., 1984), masking the expected advantage of the wild-type.

*S. epidermidis* produces a number of pro-inflammatory peptides called phenol-soluble modulins (PSMs), which are produced in a strictly *agr*-controlled manner (Mehlin et al., 1999; Yao et al., 2005). PSM-δ rapidly lyses neutrophils, supporting the idea that the peptide is of relevance to the pathogenesis of *S. epidermidis*. However, PSM-δ is expressed only at low levels by *S. epidermidis* 1457, in line with a low overall cytolytic activity of *S. epidermidis* (Cheung et al., 2010). PSM-δ is expressed only at very low levels in *S. epidermidis* 1457 biofilms as compared to planktonic cells (Wang et al., 2011a). PSM-β peptides promote *S. epidermidis* biofilm structuring and detachment *in vitro* and dissemination of infection during catheter colonization *in vivo*, thereby providing the first mechanism of biofilm detachment in *S. epidermidis* (Wang et al., 2011a).

## Outlook and future directions

Over the past two decades, significant progress has been made in our understanding of the specific pathogenic nature of *S. epidermidis* in foreign material-associated infections. A molecular picture evolved showing that *S. epidermidis* virulence is linked to biofilm formation, a phenotype that depends on a wide variety of different factors which carry distinct and tightly regulated functions during surface colonization and interactions with host immune responses. Thus, *S. epidermidis* biofilm research has reached a turning point, at which on one hand additional *in vitro* evidence for the involvement of dedicated mechanisms in surface colonization can easily be accumulated, but on the other hand the question of the *in vivo* relevance of a given factor or process certainly arises. Thus, a major future challenge will be to translate findings from highly artificial, simple *in vitro* biofilm analysis systems into complex (organ-) models that more appropriately reflect the *in vivo* infection settings. Moreover, it is of urgent importance to validate findings from *in vitro* models in relevant animal models of device infections. These approaches should involve not only state of the art molecular biology, biochemical and immunological methods but also time-resolved *in vivo* and *ex vivo* imaging technologies, allowing to create a more distinct picture of the invasive *S. epidermidis* life style in different and extremely variable environmental conditions. Using the new armament of technologies, including three-dimensional cell culture techniques and tissue engineering, efforts are necessary to study the role of *S. epidermidis* as a beneficial skin commensal more intensively.

### Conflict of interest statement

The authors declare that the research was conducted in the absence of any commercial or financial relationships that could be construed as a potential conflict of interest.
